# Supplementary material for: Antibacterial Cu or Zn-MOFs Based on the 1,3,5-Tris-(styryl)benzene Tricarboxylate
Source: Nanomaterials (Basel). 2023 Aug 9;13(16):2294. doi: 10.3390/nano13162294 (PMC10458854; doi:10.3390/nano13162294)
Supplement: Supplementary file 1 [file nanomaterials-13-02294-s001.zip › nanomaterials-2521568-supplementary.docx]

Supplemental Material

Antibacterial Cu or Zn-MOFs Based on the
1,3,5-Tris-(styryl)benzene Tricarboxylate

Sorraya Najma Kinza Lelouche ^1,2^, Laura Albentosa-González ^3,4^, Pilar Clemente-Casares ^3,4^, Catalina Biglione ^1^,
Antonio Rodríguez-Diéguez ^4^, Juan Tolosa Barrilero ^3,4^, Joaquín Calixto García-Martínez ^3,4,^*
and Patricia Horcajada ^1,5,^*

^1^ Advanced Porous Materials Unit, IMDEA Energy Institute, Av. Ramón de la Sagra 3, Mostoles,
28935 Madrid, Spain; sorraya.lelouche@imdea.org (S.N.K.L.); catalina.biglione@imdea.org (C.B.)

^2^ Escuela Internacional de Doctorado, Universidad Rey Juan Carlos, c/ Tulipan, s/n, Mostoles,
28933 Madrid, Spain

^3^ Centro Regional de Investigaciones Biomédicas (CRIB), Universidad de Castilla-La Mancha, C/Almansa 14, 02008 Albacete, Spain; laura.albentosa@uclm.es (L.A.-G.); pilar.ccasares@uclm.es (P.C.-C.);
juan.tolosa@uclm.es (J.T.B.)

^4^ Facultad de Farmacia, Universidad de Castilla-La Mancha, Av. Dr. José María Sánchez Ibáñez, s/n,
02008 Albacete, Spain; antonio5@ugr.es

^5^ Departamento de Química Inorgánica, Universidad de Granada, Av. Fuentenueva s/n, 18071 Granada, Spain

***** Correspondence: joaquinc.garcia@uclm.es (J.C.G.-M.); patricia.horcajada@imdea.org (P.H.)

**Table of content**

**S1. Optical microscopy imaging**

**S2****. FT-IR**

**S3. Crystalographic table and fitting**

**S4. Thermogravimetric analysis**

**S5. Nitrogen adsorption isotherm**

**S6. SEM**

**S7. VTPXRD**

**S8. UV-Vis Structurochemical stability test**

**S9. Structurochemical stability test**

**S10. Zeta potential**

**S11. Antibacterial activity**

**S1. Optical microscopy imaging**


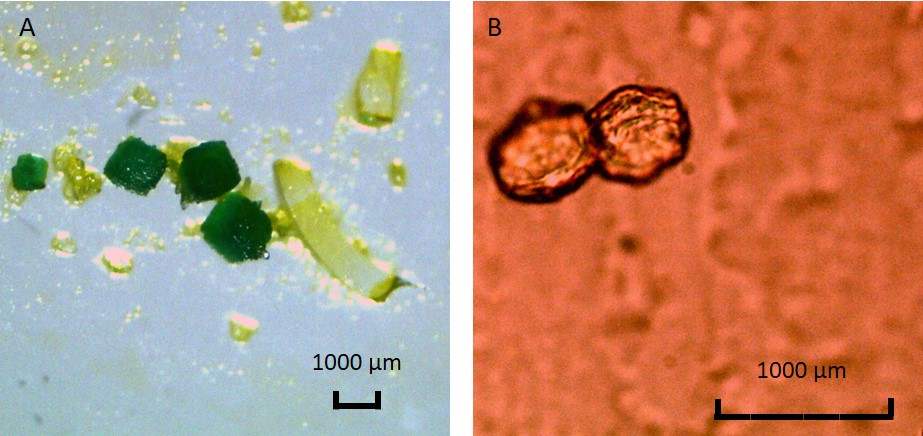


**Figure S1.** Microscopy images of: A) IEF-23 and B) IEF-24 crystals*.*

S2. FT-IR


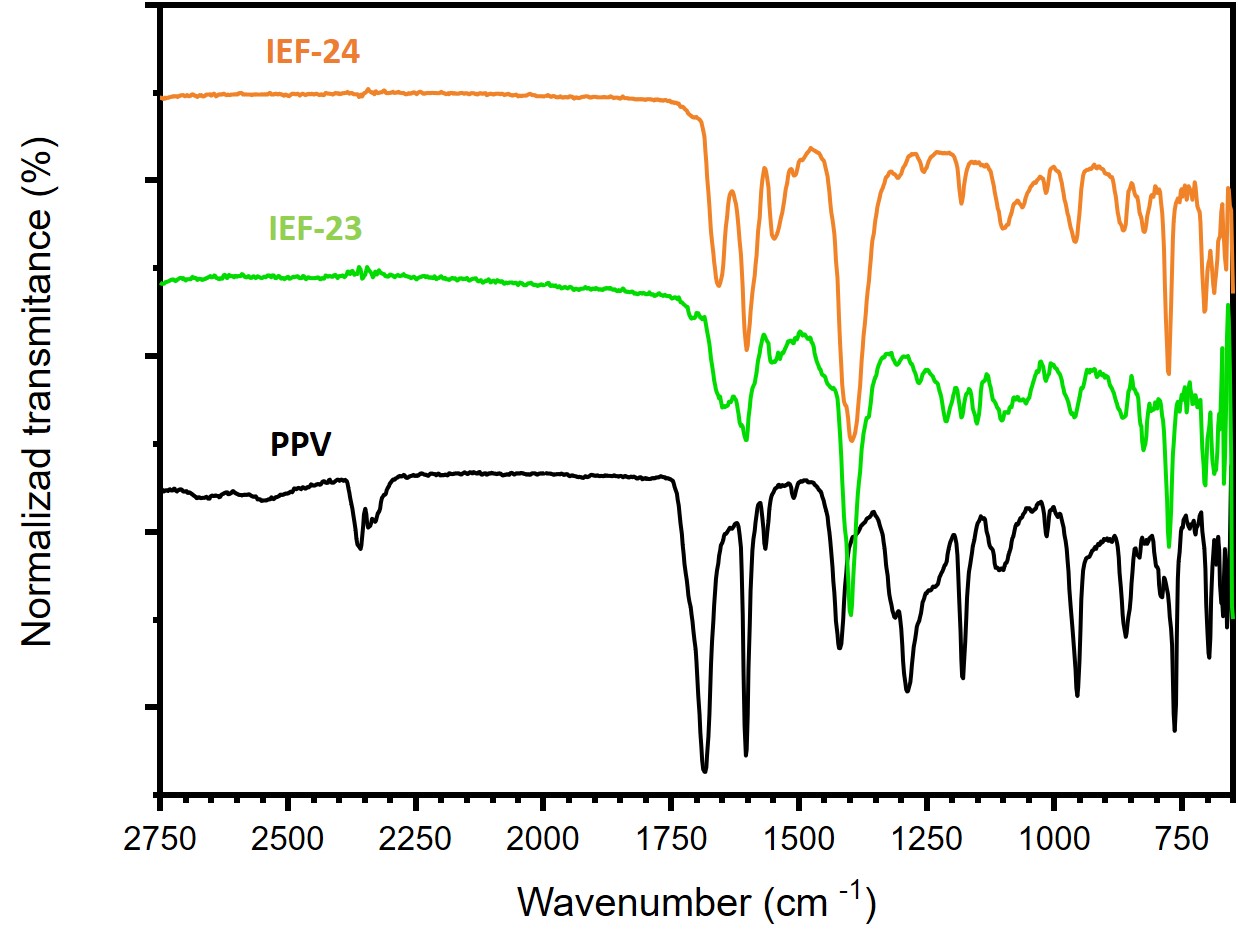


**Figure S2.** FT-IR spectra of PPV (black), IEF-23 (green) and IEF-24 (orange).

**S3.Crystalographic table and fitting**

**Table S1.** Crystallographic data summary*.*

| **Parameter** | **IEF-23**  **Exp.** | **IEF-23**  **Fitted** | **IEF-24**  **Exp.** | **IEF-24**  **Fitted** |
| --- | --- | --- | --- | --- |
| Crystal system | Cubic | Cubic | Monoclinic | Monoclinic |
| Space group | Pm3m | Pm3m | *C*2/*c* | P2/m |
| a, b, c (Å) | 29.15; 29.16; 29.30 | 29.24( 2.849 ) | 10.24;24.59;27.72 | 13.21;18.49;10.89 |
| α, β, γ (◦) | 90.21; 90.25; 90.09 | 90;90;90 | 94.74;97.74;91.97 | 90.00;90.48;90.00 |
| V (Å^3^) | 24905 | 25018(105) | 6889 | 2959 |


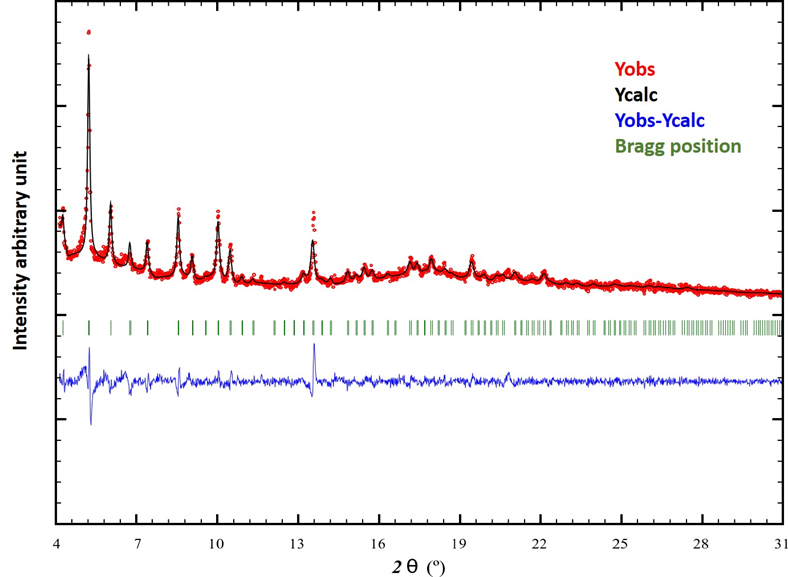


**Figure S3.** Le Bail refinement of IEF-23 (*Pm3m* (nº 221), a = 25.24(X) ; Rf-factor = *1.59%*) (λ*_Cu_*~1.5406 Å).


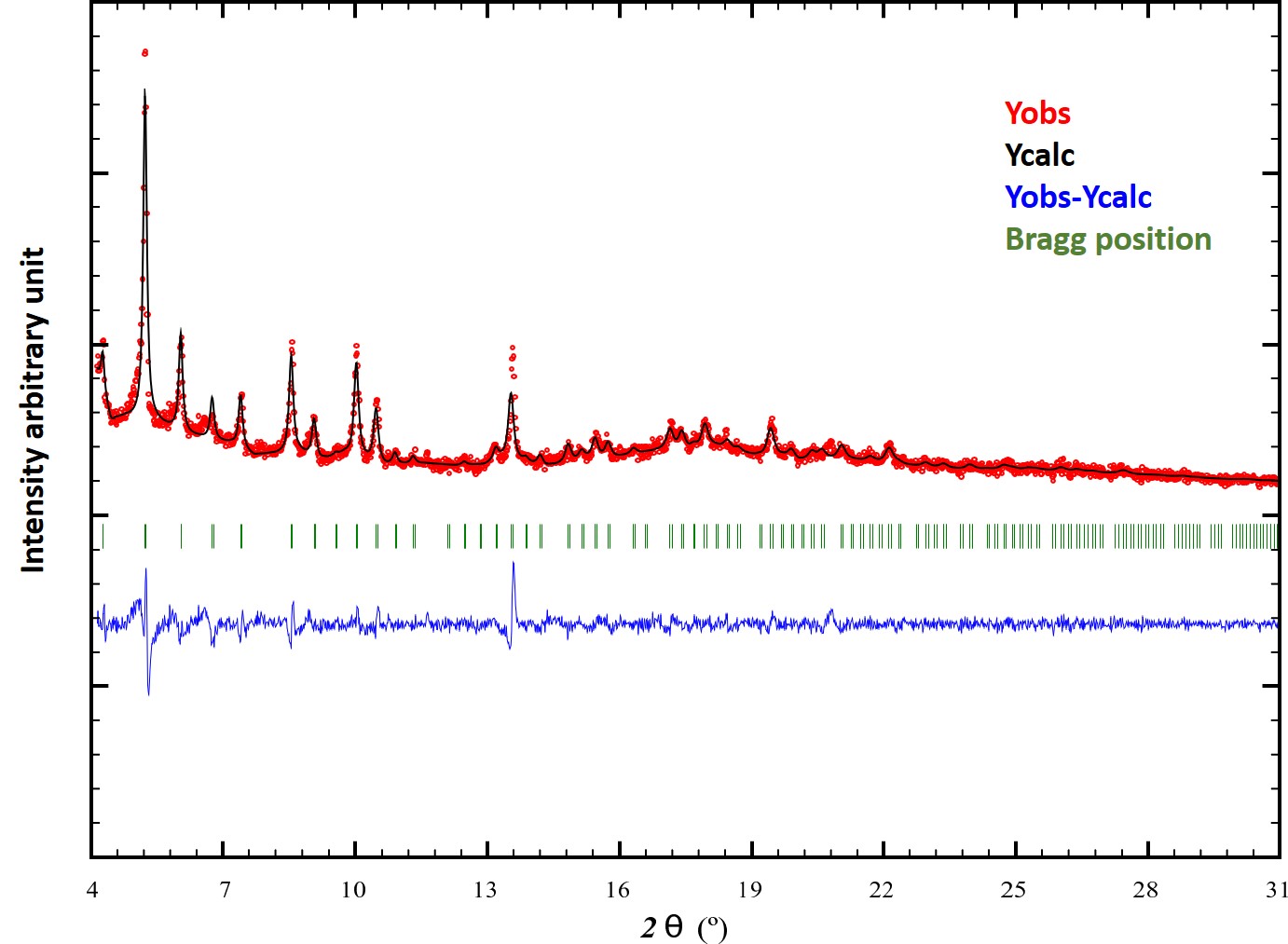


**Figure S4.** Le Bail refinement of IEF-24 (P2/m (nº 10), a = 13.21 (X) ; Rf-factor = 3.89%) (λ_Cu_~1.5406 Å).

S4. Thermogravimetric analysis


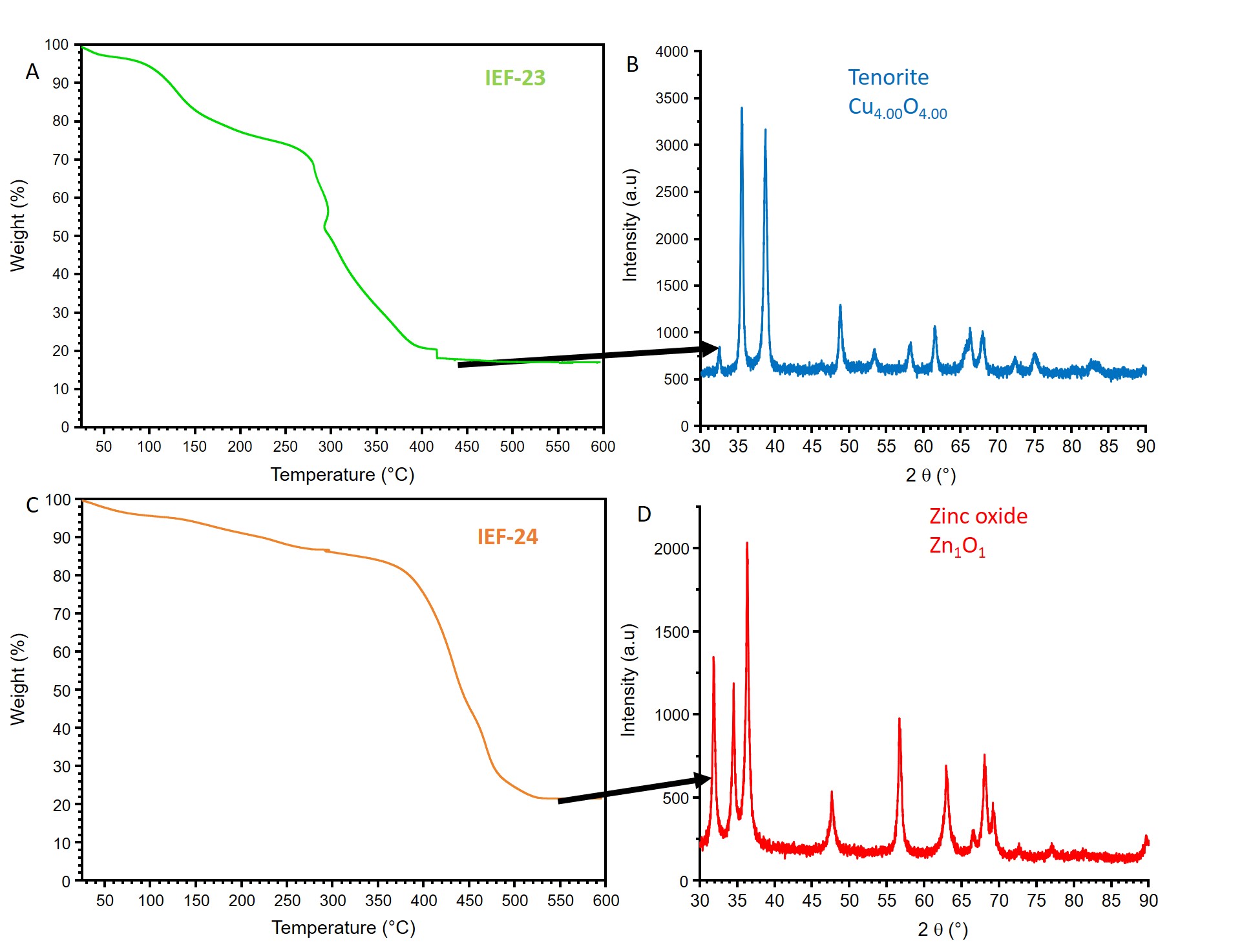


**Figure S5.** A) TGA of IEF-23; B) PXRD pattern of IEF-23 TGA residue; C) TGA of IEF-24 and D) PXRD pattern of IEF-24 TGA residue.

S5. Nitrogen adsorption isotherm


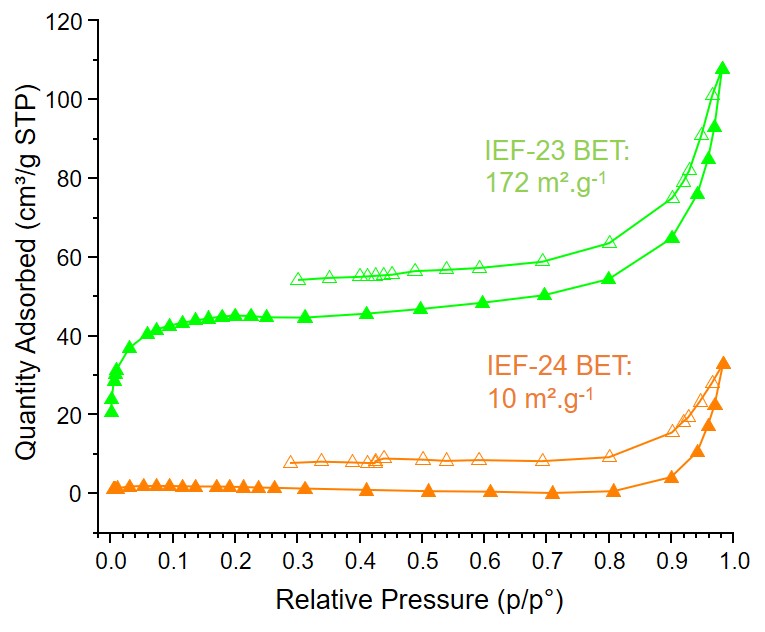


**Figure S6.** Nitrogen sorption isotherms at 77K of IEF-23 in green and IEF-24 in orange, hollow triangle for desorption and solid for adsorption*.*

**S6. SEM**


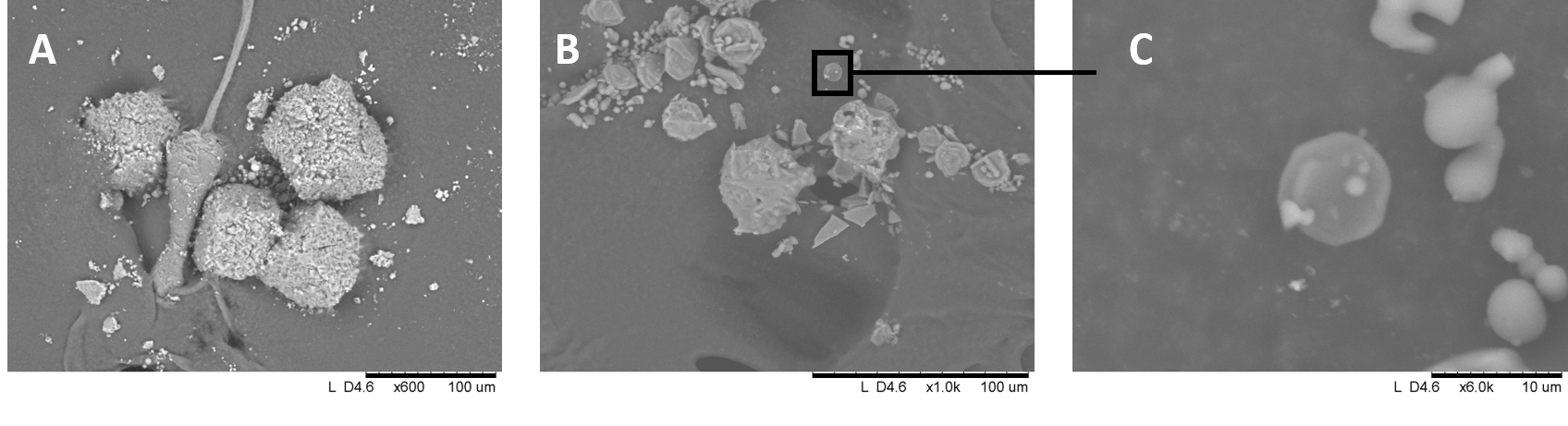


**Figure S7.** Micrographs of IEF-23 (A), and IEF-24 (B and C).

S7. VTPXRD


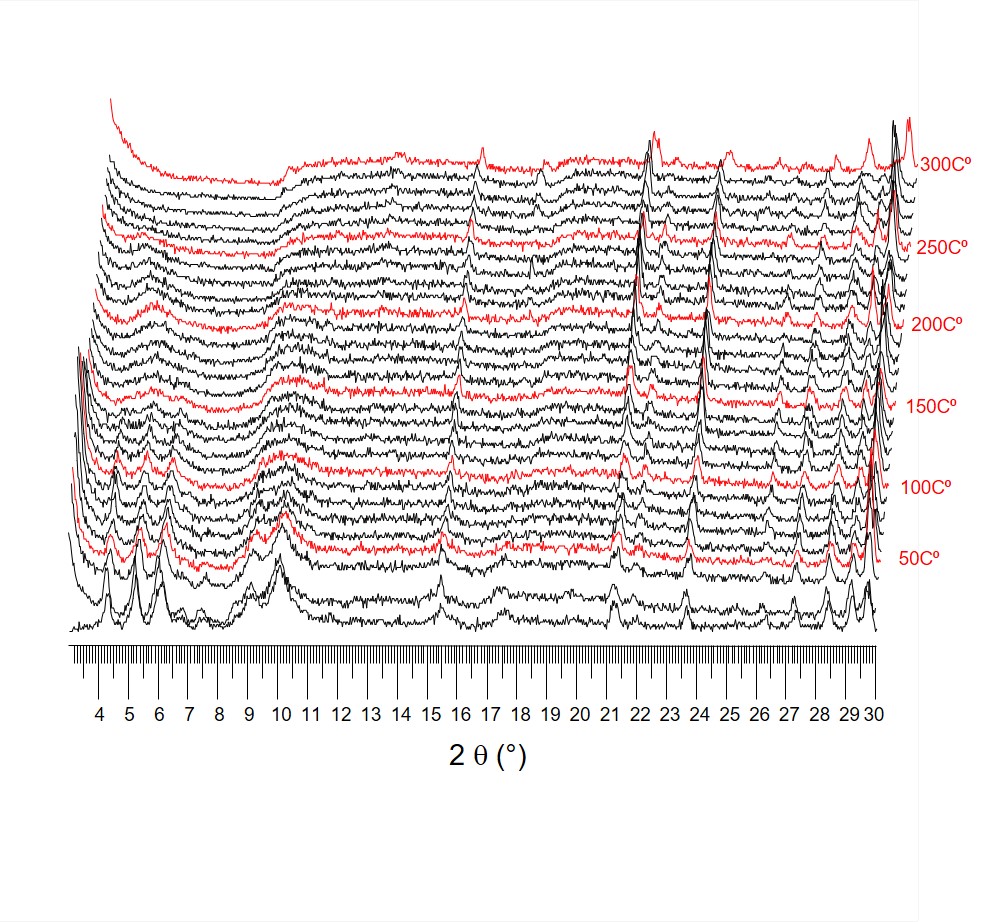


**Figure S8.** VTPXRD patterns of IEF-23 from RT to 300ºC. Each red pattern corresponds to a multiple of 50ºC.


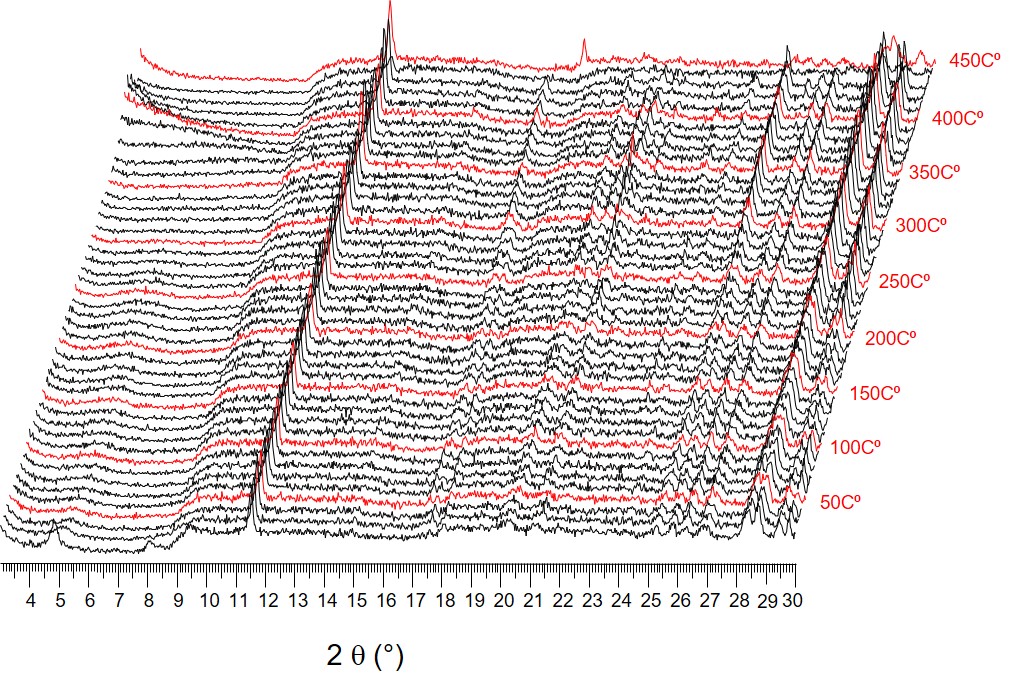


**Figure S9.** VTPXRD patterns of IEF-24 from RT to 450ºC. Each red pattern corresponds to a multiple of 50ºC.

S8. UV-Vis

**
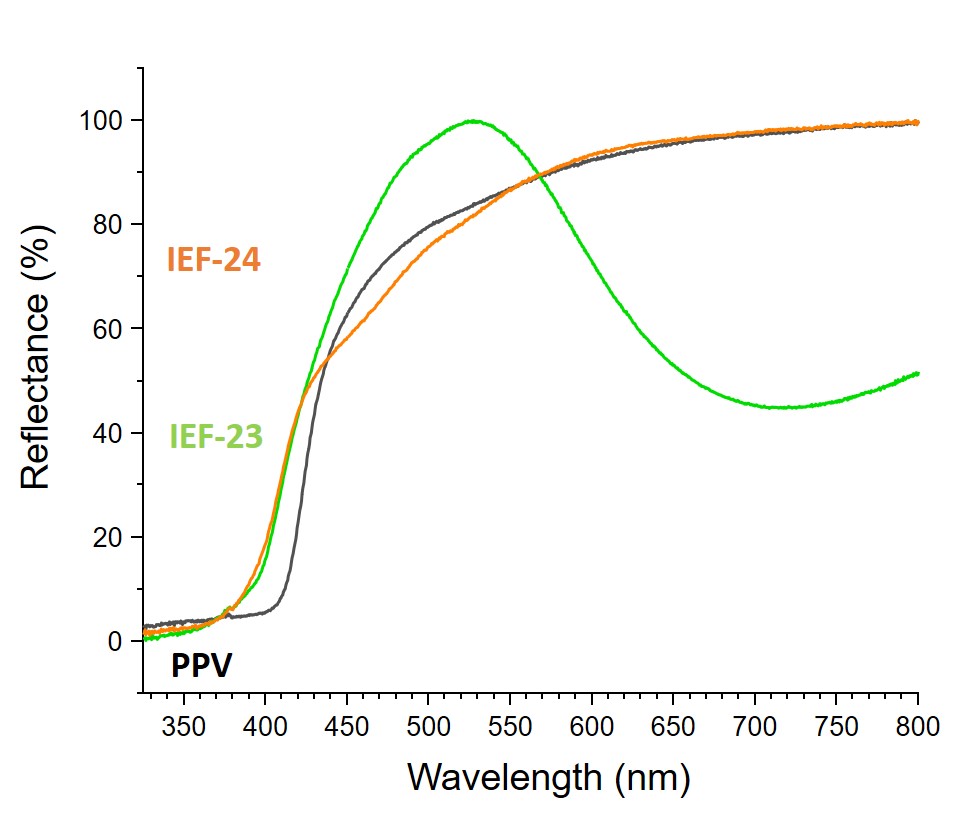
**

**Figure S10.** UV-Vis spectra in reflectance of PPV (black), IEF-23 (green) and IEF-24 (orange).


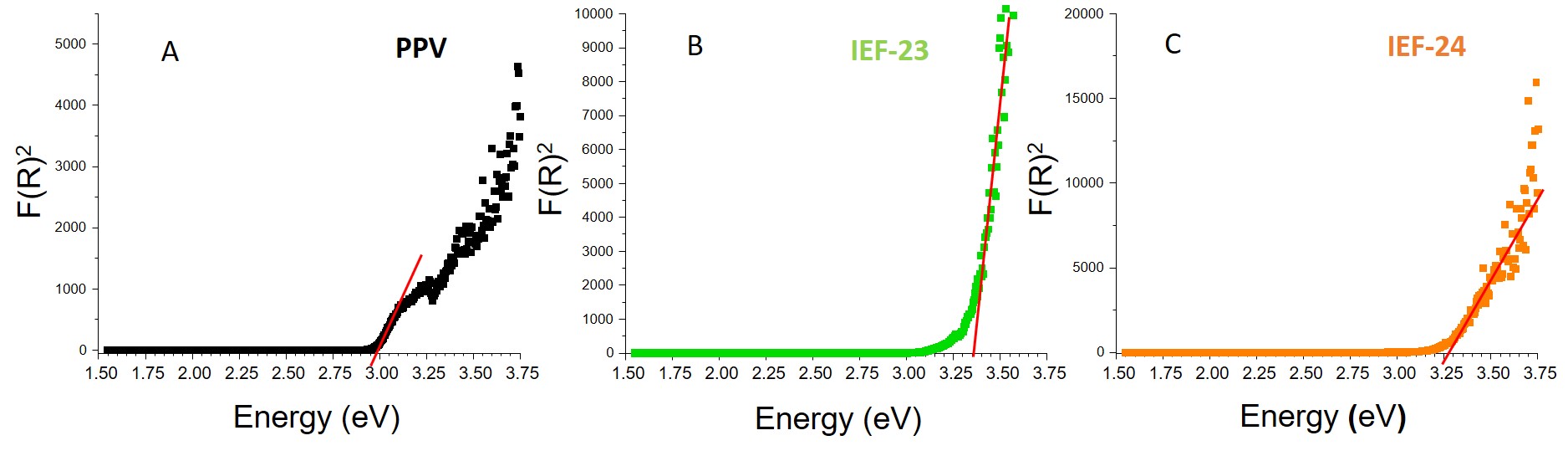


**Figure S11.** Transformed Kubelka–Munk (from diffuse reflectance spectra) function vs. energy of the excitation source of laser, of A) PPV, B) IEF-23 and C) IEF-24.

S9. Structurochemical stability test


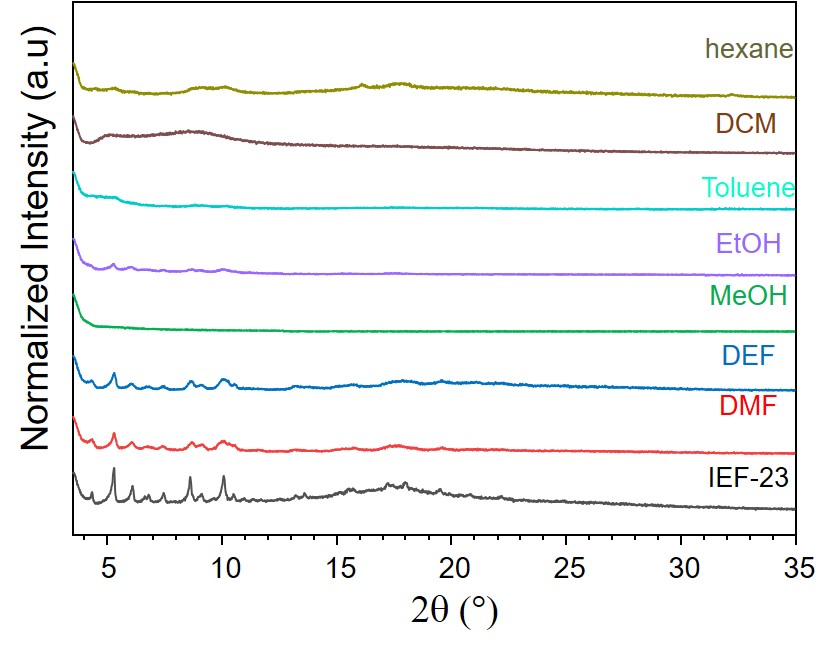


**Figure S12.** PXRD patterns of IEF-23 suspended in different media (10 mg·mL-1 for 16h, stirred 300rpm).


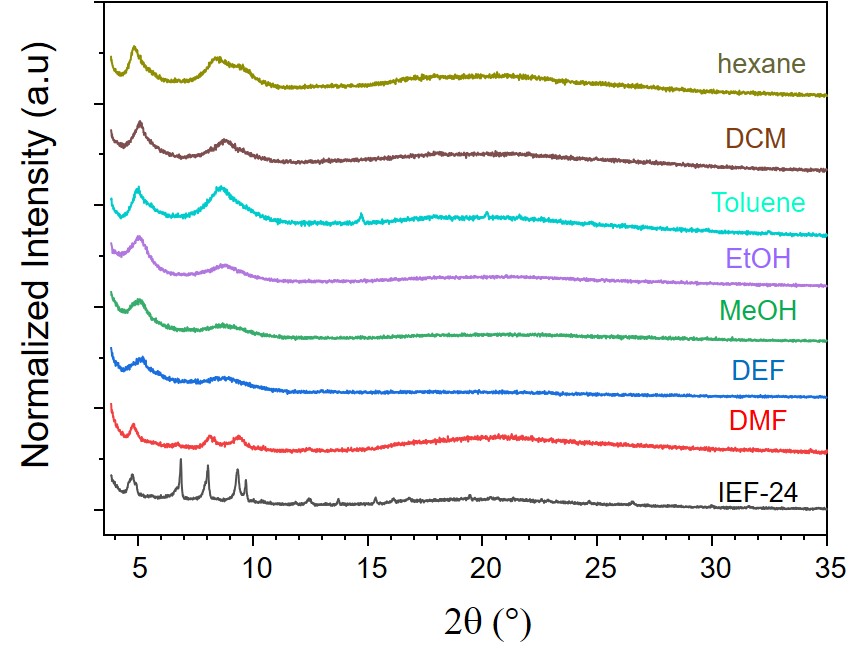


**Figure S13.** PXRD patterns of IEF-24 suspended in different media (10 mg·mL-1 for 16 h, stirred 300rpm).

**S11 Antibacterial activity**


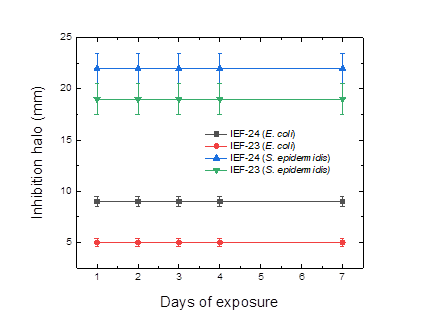


**Figure S14.** Evolution of the inhibition halo (mm; determined by KLONK) over time for IEF-24 and IEF-23 against E. coli and S. epidermidis.
